# Anxiety in children and youth with autism spectrum disorder and the association with amygdala subnuclei structure

**DOI:** 10.1177/13623613221127512

**Published:** 2022-10-22

**Authors:** Abagail Hennessy, Diane Seguin, Susana Correa, Jianan Wang, Julio C Martinez-Trujillo, Rob Nicolson, Emma G Duerden

**Affiliations:** 1 Western University, Canada; 2 The University of Western Ontario, Canada

**Keywords:** amygdala, autism spectrum disorders, behavior, human, MRI, social

## Abstract

Autism spectrum disorder (ASD) is clinically characterized by social and communication difficulties as well as repetitive behaviors. Many children with ASD also suffer from anxiety, which has been associated with alterations in amygdala structure. In this work, the association between amygdala subnuclei volumes and anxiety was assessed in a cohort of 234 participants (mean age = 11.0 years, SD = 3.9, 95 children with ASD, 139 children were non-autistic). Children underwent magnetic resonance imaging. Amygdala subnuclei volumes were extracted automatically. Anxiety was assessed using the Screen for Child Anxiety Related Disorders, the Child Behavior Checklist, and the Strength and Difficulties Questionnaire. Children with ASD had higher anxiety scores relative to non-autistic children on all anxiety measures (all, p < 0.05). Anxiety levels were significantly predicted in children with ASD by right basal (right: B = 0.235, p = 0.002) and paralaminar (PL) (B = −0.99, p = 0.009) volumes. Basal nuclei receive multisensory information from cortical and subcortical areas and have extensive projections within the limbic system while the PL nuclei are involved in emotional processing. Alterations in basal and PL nuclei in children with ASD and the association with anxiety may reflect morphological changes related to in the neurocircuitry of anxiety in ASD.

**Lay abstract:**

Autism spectrum disorder (ASD) is clinically characterized by social communication difficulties as well as restricted and repetitive patterns of behavior. In addition, children with ASD are more likely to experience anxiety compared with their peers who do not have ASD. Recent studies suggest that atypical amygdala structure, a brain region involved in emotions, may be related to anxiety in children with ASD. However, the amygdala is a complex structure composed of heterogeneous subnuclei, and few studies to date have focused on how amygdala subnuclei relate to in anxiety in this population. The current sample consisted of 95 children with ASD and 139 non-autistic children, who underwent magnetic resonance imaging (MRI) and assessments for anxiety. The amygdala volumes were automatically segmented. Results indicated that children with ASD had elevated anxiety scores relative to peers without ASD. Larger basal volumes predicted greater anxiety in children with ASD, and this association was not seen in non-autistic children. Findings converge with previous literature suggesting ASD children suffer from higher levels of anxiety than non-autistic children, which may have important implications in treatment and interventions. Our results suggest that volumetric estimation of amygdala’s subregions in MRI may reveal specific anxiety-related associations in children with ASD.

## Introduction

Autism spectrum disorder (ASD) is a neurodevelopmental disorder which is clinically characterized by difficulties in social interaction and communication, repetitive behaviors, and restricted interests. Heightened levels of anxiety are common in youth with ASD compared with non-autistic individuals, with up to 85% of children with ASD reporting symptoms of anxiety and nearly 40% of children with ASD diagnosed with at least one comorbid anxiety disorder such as generalized anxiety disorder or social anxiety (van Steensel et al., 2012). Moreover, there is a current need to identify those children and adolescents with ASD who have high likelihood of developing anxiety. The reciprocal nature of anxiety and poor social skills may altogether worsen a child’s peer interactions, relationships, and quality of life (Bellini, 2004; van Steensel et al., 2011). The neural mechanisms underlying anxiety in children and adolescents with ASD are still poorly understood.

As the amygdala is involved in processing socio-emotional stimuli and evaluating stimulus salience (Herrington et al., 2017; Truitt et al., 2007) considerable research has focused on the structural development of the amygdala in children with ASD (Li et al., 2019; Munson et al., 2006; Nordahl et al., 2012). Studies have reported larger total amygdala volumes present in young children later diagnosed with ASD (Avino et al., 2018), with this early overgrowth detectable in children as young as 6 months of age (Li et al., 2019). Amygdala overgrowth may be limited to the infancy and early childhood developmental period (Shen et al., 2022) as smaller amygdala volumes by late adolescence and early adulthood have been reported in autistic participants with findings suggesting that social impairment severity is associated with smaller adult amygdala volumes (Nacewicz et al., 2006).

Fewer studies have examined the volume of this structure in relation to anxiety in ASD, and current findings are mixed. It is important to note that anxiety can be measured in a variety of ways, and anxiety scores may be collected via clinical interviews with participants, parent reports, and child self-reports. Larger amygdala volumes were associated with increased anxious and depressed parent-reported symptoms in ASD children (Juranek et al., 2006) while in a cross-sectional study, children with high parent-reported anxiety symptoms and ASD displayed smaller right amygdala volumes than non-autistic children (Herrington et al., 2017). A recent study with children and young adolescents with ASD indicated no association between parent-reported anxiety and amygdala volumes (Yarger et al., 2021). Another study included longitudinal assessments of anxiety and amygdala volumes in children (Andrews et al., 2022). Clinical anxiety was associated with larger amygdala volumes in children with autism; however, slower growth in the amygdala was reported in children with autism who had autism-specific anxiety. Enlarged amygdala volumes have also been reported in non-autistic children and adults with self-reported and clinician-reported anxiety (De Bellis et al., 2000; Machado-de-Sousaetal., 2014).

The amygdala is a heterogeneous structure composed of subnuclei which differ in their input and output regions, and associated functions. These subregions include the lateral, basal, accessory basal, central, medial, anterior amygdaloid area, cortical amygdala, corticoamygdaloid transition area, and paralaminar nucleus. The lateral nucleus receives sensory input from the thalamus, cortex and hippocampus, and then projects to the basal and accessory basal nuclei. Together, the lateral, basal, and accessory basal nuclei form the basolateral amygdala (BLA) complex, which connects to the prefrontal cortex (PFC), and the orbitofrontal cortex (OFC). The BLA is involved in predicting and coordinating responses to sensory and social stimuli (Truitt et al., 2007). The BLA is believed to contribute to anxiety as it provides information for the hypothalamic-pituitary-adrenal (HPA) stress response system. In ASD, larger BLA volumes were evident when compared with non-autistic children, and increased volume of the BLA in children with ASD was associated with increased challenges with social skills (Seguin et al., 2021). It has been shown that activation of BLA projections to the medial prefrontal cortex (mPFC) results in heightened anxiety responses in rodents, while inhibition of these projections results in dampened anxiety behaviors (Felix-Ortiz et al., 2016). BLA dysfunction is thought to underlie some of the social behavioral challenges seen in some autistic individuals, such as difficulties interpreting and appropriately responding to social cues, and an avoidance of social stimuli and interactions (Nacewicz et al., 2006; Sinha et al., 2015; Truitt et al., 2007).

The central (Ce) nucleus acts as the primary output region connected to subcortical and cortical regions and is heavily involved in processing and responding to aversive or threatening stimuli (Fadok et al., 2018; LeDoux et al., 1990). As well as projecting to the periaqueductal gray area and the hypothalamus, the Ce nucleus also releases corticotropin releasing factors (CRF), which are involved in maintaining the body’s stress responses via the HPA system (Keen-Rhinehart et al., 2009). The Ce nucleus has strong connections to the bed nucleus of the stria terminalis and these regions together form defensive responses to threatening stimuli (Fox et al., 2008). Ce nucleus growth in autistic children was recently found to relate to degree of restrictive and repetitive behaviors (Seguin et al., 2021), which themselves are associated with anxiety (Jiujias et al., 2017; Rodgers et al., 2017).

The medial nucleus contributes to typical social behaviors in humans (Haller, 2018; Knickmeyer et al., 2014). Results from animal studies support the role of the medial nucleus in behavioral responses to environmental stimuli (Vinkers et al., 2010). In children with ASD, increased growth in the medial nucleus was associated with both social and communication challenges (Seguin et al., 2021). The anterior amygdaloid area (AAA) and the cortical amygdala (CoA) both receive olfactory information and are believed to contribute to fear conditioning and defensive behaviors in animals (Cádiz-Moretti et al., 2016) as well as social communication in humans (Bzdok et al., 2013).

The corticoamygdaloid transition area (CAT) and paralaminar nucleus (PL) have both been linked to affective disorders, such as schizophrenia (Barth et al., 2021) with smaller volumes associated with the disorder (Tesli et al., 2020). The PL nucleus contains immature neurons which remain immature into adulthood, and it is believed these neurons may migrate to other nuclei across development (Sorrells et al., 2019). The PL has connections with the Ce nucleus and is thought to contribute to emotional responses (LeDoux, 2007).

Given the complexity of the organization of the input and output subnuclei of the amygdala, and their widespread roles integrating information from separate modalities, it is critical to determine their morphology in relation to anxiety in children with ASD. The input nuclei of the BLA are of particular interest in relation to ASD diagnosis and anxiety due to their “gate-keeping” role in determining relevant social stimuli, and providing input to higher order cortices to shape social behavior (Sinha et al., 2015). As previous work has indicated that children with ASD have heightened anxiety attributed to alterations in whole amygdala volumes, we aimed to examine these associations in relation to the volumes of the amygdala subnuclei in a large heterogeneous cohort of children and adolescents with ASD compared with non-autistic children and adolescents.

Other key variables which may also influence both anxiety and amygdala growth must be considered. Socioeconomic status (SES) is known to be an important predictor of brain development and may thus influence brain volume differences among child populations. Specifically, significant variability in brain surface area has been found among children from lower income families (Brito & Noble, 2014; Merz et al., 2018; Noble et al., 2005, 2006; Piccolo et al., 2016; Ursache et al., 2016). Children from lower SES households are more also likely to develop anxiety and depression later in life than children from higher SES households (Kinge et al., 2021; Merz et al., 2018). Volumetric growth in the amygdala occurs during child development through to adolescence (Schumann et al., 2004), and patterns of amygdala subnuclei growth have been found to differ between autistic and non-autistic children and youth at different developmental stages (Courchesne et al., 2011; Mosconi et al., 2009; Schumann et al., 2009). When examining the effects of sex on amygdala growth, the current literature in non-autistic participants provides mixed findings, with reports of larger volumes in males than females (Brierly et al., 2002; Giedd et al., 1996), and or no volumetric sex-based differences (Liao et al., 2014). Recent work has reported proportional volumes of some amygdala subregions change during development in adolescent males, but not females (Campbell et al., 2021). In youth with ASD, associations between amygdala subnuclei growth and scores on the Autism Diagnostic Observation Schedule (ADOS) were significantly influenced by sex (Seguin et al., 2021).

We used multiple measures to capture anxiety experienced by participants. It has been suggested that no single factor can accurately capture a child’s experiences with anxiety (White et al., 2009). We analyzed four different anxiety measures: Screen for Child Anxiety Related Disorders–Parent Report (SCARED-P), Screen for Child Anxiety Related Disorders–Self-Report (SCARED-SR), Child Behavior Checklist–Anxious Depressed subscale (CBCL-AD), and the Strengths and Difficulties Questionnaire (SDQ-Internalizing). Self-report and parent report measures were utilized in favor of clinical interviews to address the present aim of quantifying anxiety symptoms in the sample, as opposed to assessing the presence of anxiety disorders (Peris & Rozenman, 2019). Inclusion of a self-report measure such as the SCARED-SR is of importance as anxiety is an internalizing disorder and as such, a child’s report may more accurately capture their personal experience better than the interpretation of an external rater, such as a parent (Tandon et al., 2009). In ASD particularly, where some children may lack the appropriate insight or ability to report their own anxiety symptoms, parent reports are necessary to include in anxiety assessments. Parents or guardians may be able to provide information on changes in behavior associated with anxiety, or transient behaviors which may not be evident during a clinician’s assessment. However, parents may encounter difficulty in differentiating between ASD and anxiety symptoms (van Steensel et al., 2012). These differences are evident in some measures of anxiety themselves (Vasa et al., 2016). Our central hypothesis was that children and youth with ASD would have increased anxiety compared with non-autistic children and youth and that these differences would be associated with alterations in amygdala subnuclei volumes. As this is a preliminary study, we had no *a priori* hypotheses regarding which amygdala subnuclei would exhibit volumetric differences between groups. Better understanding of the volumetric differences in the amygdala’s subnuclei may reveal important patterns of structural atypicalities in children and youth with ASD who experience anxiety.

## Methods

### Participants

The participants were recruited through the Healthy Brain Network Biobank. A total of 234 children and youth participated in the current study. Participants ranged in age from 5.0 to 20.6 years of age (M = 11.0, SD = 3.9) (Alexander et al., 2017). Participants were recruited from the community based on advertisements. A total of 95 participants had an ASD diagnosis (77 males, 18 females), and 139 participants were non-autistic (68 males and 71 females). Participant demographics are displayed in Table 1.

**Table 1.** Participant demographics.

Characteristic	ASD	Non-autistic	Chi-square/*t* value	p value
*N* (%)	95 (41%)	139 (59%)		
Male sex (%)	77 (81%)	68 (49%)	21.8	<0.001
Age [SD]	11.7 [4.0]	10.5 [3.7]	2.3	0.03
BSMSS [SD]	48.2 [14.6]	50.7 [13.5]	1.3	.2
ASSQ [SD]	18.8 [10.3]	2.7 [4.4]	16.1	<0.001
SCARED-P TOTAL [SD]	18.3 [13.2]	9.3 [7.8]	6.1	<0.001
SCARED-SR TOTAL [SD]	22.7 [15.9]	22.7 [15.9]	2.9	<0.001
CBCL-AD [SD]	61.3 [9.5]	53.0 [4.9]	8.3	<0.001
SDQ-Internalizing [SD]	7.0 [3.3]	2.8 [2.7]	10.2	<0.001
Whole amygdala vol [SD]	3539.5 [436.7]	3463.5 [410.0]	1.4	0.176

Means and standard deviations of the ages SCARED scores, as well as the number of males and females for both the ASD and the TD groups. Probability values provide results using a *t* test for continuous measures and chi-square tests for categorical measures. ASD: autism spectrum disorder; BSMSS: Barratt Simplified Measure of Social Status; ASSQ: Autism Spectrum Screening Questionnaire; SCARED-P: Screen for Child Anxiety Related Disorders–Parent Report; SCARED-SR: Screen for Child Anxiety Related Disorders–Self-Report; CBCL-AD: Child Behavior Checklist–Anxious Depressed Subscale; SDQ: Strengths and Difficulties Questionnaire Internalizing subscale; TD: typically developing.

All participants were screened for medical or cognitive/behavioral challenges that could affect participation such as chronic epilepsy, brain injury or IQ < 66 (for full exclusion criteria, see Alexander et al., 2017). Participants 18 years of age or older provided written consent. Written consent was obtained from legal guardians for all participants under the age of 18, and written assent was obtained from the participant (Alexander et al., 2017). This study was approved by the local Institutional Review Board. There was no community involvement in the reported study. The data were accessed in September 2020.

### Demographics and diagnostic assessments

Parents completed demographic questions, which included the Barratt Simplified Measure of Social Status (BSMSS) to measure socioeconomic status. This measure of social status is based on marital status, employment status, educational attainment, and occupation (Barratt, 2006).

All participants were administered the Kiddie Schedule for Affective Disorders and Schizophrenia (KSADS). Participants that exhibited additional clinical behaviors consistent with ASD received follow-up assessments using the ADOS-2 and the ADI-R to reach a primary diagnosis of ASD. Participants who did not meet diagnostic criteria on the KSADS and who did not display any other clinically relevant behaviors were allocated to the typically developing, non-autistic comparison group.

#### Anxiety measures

Parents completed the parent version of the SCARED (SCARED-P), CBCL-AD, and the Strength and Difficulties Questionnaire–Internalizing symptoms subscale (SDQ-Internalizing), while children and adolescents completed the self-report version of the SCARED (SCARED-SR). The SCARED scores children’s anxiety-related behaviors on a scale from 0 (not true) to 2 (very true). The CBCL measures the frequency of children’s behaviors from 0 (absent) to 2 (occurs often). Higher scores on the anxious/depressed subscale relate to higher anxiety behaviors. The SDQ asks whether positive or negative attributes are true of the child, with a scale from 0 (not true) to 2 (certainly true). The internalizing subscale of the SDQ includes questions, which captures emotional and peer-related feelings.

### Magnetic resonance imaging protocol

MRI data were collected on a 3 T Siemens scanner using a Siemens 32-channel head coil. High-resolution T1-weighted MPRAGE structural images were acquired in 224 sagittal slices (repetition time [TR] = 2500 ms, Echo Time [TE] = 3.15 ms, resolution = 0.8 × 0.8 mm^2^). Participant MRI scans were collected at three different data collection sites: Staten Island, Rutgers University, and the CitiGroup Cornell Brain Imaging Center (Manhattan). The scanning protocol at the three different data collection sites underwent rigorous testing to minimize scanner effects, whereby 20 participants were scanned twice at the three data collection sites (Alexander et al., 2017). During the participants’ first visit and initial assessments, they were exposed to a mock MRI experience prior to undergoing scanning.

### Cortical and subcortical segmentation

**Figure 1.**
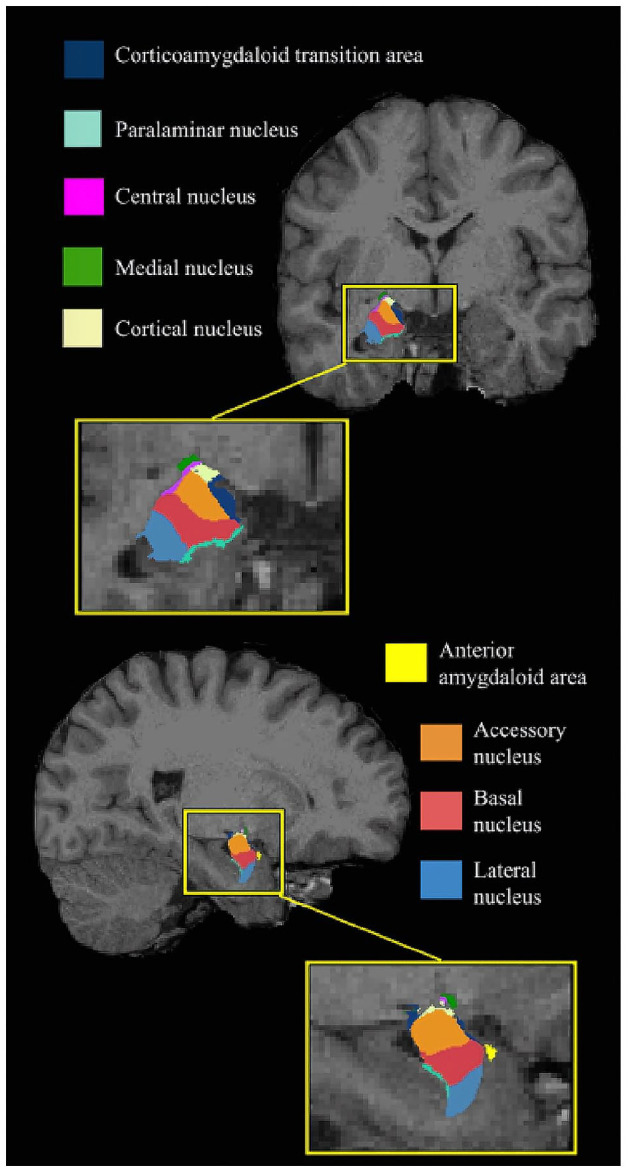
Amygdala atlas registered to a T1-weighted image obtained in a non-autistic individual.

The total cerebral volumes, amygdalae, and its subnuclei were automatically segmented using FreeSurfer version 6.0 (http://surfer.nmr.mgh.harvard.edu). All images were visually inspected for the presence of distortions including motion artifacts. The automatic regional segmentation by the FreeSurfer pipeline was visually qualified by two authors on the graphic interface FreeView, available with the Freesurfer suite of tools (http://surfer.nmr.mgh.harvard.edu/). No scans were excluded from the analyses. The amygdala was segmented into the lateral, basal, accessory basal, central (Ce), corticoamygdaloid transition area (CTA), medial, cortical, paralaminar nuclei, and the anterior amygdaloid area (AAA, Figure 1). Individual amygdala subnuclei segmentations were further visually inspected using ITK-SNAP (http://www.itksnap.org/) (Seguin et al., 2022). Volumes from the FreeSurfer automatic segmentation of the amygdala subnuclei were extracted into a spreadsheet using an in-house software available on the Developing Brain Lab github website: (https://github.com/DevelopingBrainLab/amygdala_segmentation_Freesurfer).

#### Statistical analysis

All statistical analyses were conducted using SPSS (version 27, Statistical Package for the Social Sciences, Chicago, IL). A generalized linear model (GLM) with an identity link function was run for each anxiety measure (SCARED-P, SCARED-SR, CBCL-AD, SDQ-Internalizing subscales; dependent variables) to compare anxiety scores between non-autistic and ASD participant groups (independent variable), adjusting for age, biological sex (reference group = males), data collection site, and SES. Anxiety scores were similarly distributed across data collection sites. As four anxiety measures were being investigated, a Bonferroni correction was used with the alpha value set at p = 0.0125 (0.05/4 = 0.0125).

To determine whether amygdala subnuclei volumes (dependent variable) differed between the diagnostic groups (independent variable), GLMs were performed, adjusting for age, biological sex, SES, data collection site, and total cerebral volume (TCV). As there were 18 amygdala subnuclei examined, nine per hemisphere, the adjusted alpha level was p = 0.002.

The association of amygdala subnuclei volumes with anxiety scores (dependent variables) was assessed in children with ASD compared with non-autistic children in a series of GLMs, with participant group and amygdala subnuclei volumes as independent variables, adjusting for biological sex, age, SES, data collection site, and TCV, with an adjusted alpha level of p = 0.0125, to control for multiple comparisons. Whole amygdala volumes were also assessed in relation to anxiety measures adjusting for the same covariates. Finally, a GLM was run to test for significant interaction effects between diagnostic groups and subnuclei volumes in predicting anxiety scores, and including significant covariates identified in the main models.

## Results

### Anxiety in children and youth with and without ASD

The anxiety scores on the SCARED-P were compared between non-autistic and ASD groups (Table 2). The two diagnostic groups differed significantly on the total SCARED-P scores, with the ASD group demonstrating higher scores relative to the non-autistic group (B = 10.373, p < 0.001). No other variables were significant in the model. When examining SCARED-SR scores, there was a significant association with diagnosis (B = 9.211, p = 0.011), with ASD participants exhibiting higher scores than non-autistic participants (Table 2) and no significant associations with other variables. When examining significant contributors to SDQ-Internalizing scores, diagnosis was significant, with higher anxiety evident in ASD participants (B = 4.60, p < 0.001). For CBCL-AD scores, ASD participants displayed higher scores than non-autistic participants (B = 6.72, p < 0.001). Sex, age, SES, and data collection site were not significantly associated with CBCL-AD scores.

**Table 2.** Anxiety scores.

Predictor variables	SCARED-P	SDQ-Internalizing	SCARED-SR	CBCL-AD
Diagnosis	B = 10.37, p < 0.001*	B = 4.60, p < 0.001*	B = 9.21, p = .011*	B = 6.72, p < 0.001*
Age	B = 0.043, p = .836	B = 0.123, p = .031	B = −0.692, p = .028	B = 0.005, p = .974
Sex	B = −3.23, p = .044	B = −0.93, p = .028	B = −4.83, p = .026	B = −1.39, p = .195
Site	B = 2.88, p = .077	B = 0.91, p = .038	B = 2.74, p = .222	B = 0.350, p = .752
SES	B = −0.094, p = .09	B = −0.034, p = .022	B = −0.137, p = .075	B = < 0.001, p = .993

Results from generalized linear models investigating the association between Anxiety Scores (SCARED-P, SDQ-Internalizing, SCARED-SR, and CBCL-AD: dependent variables) and diagnosis (independent variable) with age, biological sex, testing site, and SES as covariates. Beta values and p values are shown. The alpha level has been adjusted for multiple comparisons. SCARED-P: Screen for Child Anxiety Related Disorders–Parent Report; SDQ: Strengths and Difficulties Questionnaire Internalizing subscale; SCARED-SR: Screen for Child Anxiety Related Disorders–Self-Report; CBCL-AD: Child Behavior Checklist–Anxious Depressed Subscale; SES: socioeconomic status.

* p < .0125.

A total of 12 autistic participants had a secondary anxiety or a related anxiety disorder diagnosis (e.g., social anxiety, obsessive compulsive disorder). To determine whether the anxiety scores of these participants were unduly influencing the results of the ASD group, we ran the anxiety analyses with the data from these participants omitted. No differences were found following the removal of the data from these participants. The ASD participants without a comorbid diagnosis of anxiety also had significantly higher anxiety, on all four anxiety, measures compared with non-autistic participants. In turn, we retained these participants for all analyses.

### Between-group differences in amygdala subnuclei volumes

**Figure 2.**
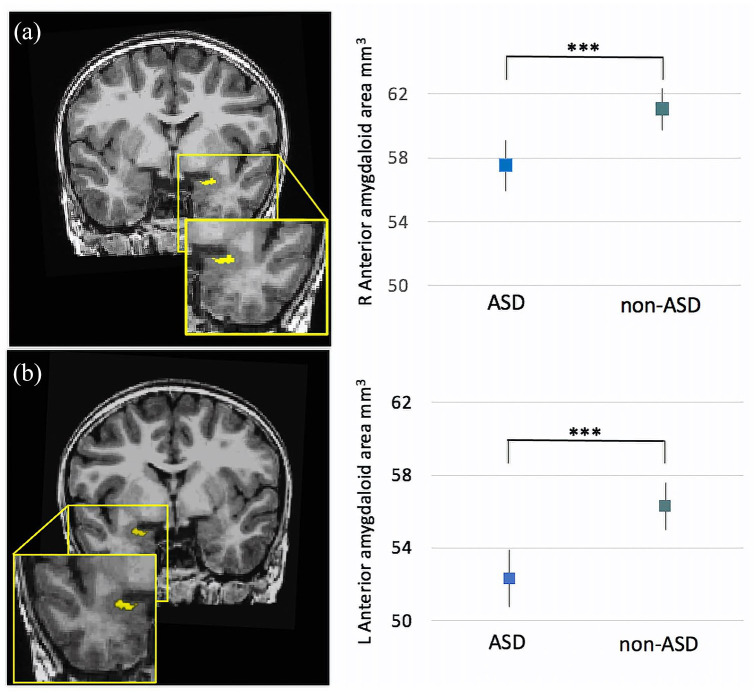
(a) Estimated marginal means of the AAA volumes for ASD and non-ASD groups. Children with ASD had smaller right AAA volumes compared with the non-ASD group (*B* = −3.434, p < 0.001). (b) Children with ASD had smaller left AAA volumes compared with the non-ASD group (*B* = −4.217, p < 0.001). Error bars represent 95% confidence intervals. ***p < 0.001.

Right AAA volumes were found to be significantly smaller in ASD participants than non-autistic participants (B = −3.54, p < 0.001, Figure 2a), as were left AAA volumes (B = −4.22, p < 0.001) (Figure 2bTable 3).

**Table 3.** Result of generalized linear models for amygdala subnuclei volumes in relation to ASD diagnosis.

Predictor variables	L Lateral	L Basal	L Accessory Basal	L AAA
Diagnosis	B = −7.691, p = .144	B = −13.059, p = .138	B = 0.504, p = .884	B = −4.217, p < 0.001*
Age	B = 3.569, p < 0.001*	B = 5.429, p < 0.001*	B = 2.746, p < 0.001*	B = 0.225, p = .087
Sex	B = 13.582, p = .019	B = 22.232, p = .021	B = 6.926, p = .067	B = 2.207, p = .056
Site	B = −2462, p = .644	B = −7.233, p = .417	B = −1.698, p = .627	B = −0.708, p = .507
SES	B = 0.230, p = .187	B = −0.010,p = .973.	B = 0.290, p = .011	B = 0.035,p = .316
TCV	B < 001, p < 0.001*	B < 0.001, p < 0.001*	B < 0.001, p < 0.001*	B < 0.001, p < 0.001*
Predictor variables	L Central	L Medial	L Cortical	L CAT
Diagnosis	B = −0.551, p = .633	B = −0.251, p = .752	B = 0.459, p = .441	B = 1.229, p = .623
Age	B = 0.438, p = .002*	B = 0.301, p = .002*	B = 0.309, p < 0.001*	B = 1.984p < 0.001*
Sex	B = 3.407, p = .007	B = 0.882, p = .311	B = 0.621, p = .343	B = 4.940, p = .072
Site	B = −1.278, p = .274	B = 0.251, p = .755	B = 0.240, p = .691	B = −1.538, p = .544
SES	B = 0.017,p = .664	B = −0.022,p = .410	B = 0.024,p = .230	B = 0.129, p = .119
TCV	B < 0.001, p < 0.001	B < 0.001, p < 0.001*	B < 0.001, p < 0.001*	B < 0.001, p < 0.001*
Predictor variables	L Paralaminar	R Lateral	R Basal	R Accessory Basal
Diagnosis	B = −0.626, p = .344	B = −16.902, p = .038	B = −12.607, p = .019	B = −0.230, p = .949
Age	B = 0.300, p < 0.001*	B = 5.575, p < 0.001*	B = 4.244, p < 0.001*	B = 3.207, p < 0.001*
Sex	B = 2.321, p = .001*	B = 29.356, p = .001	B = 9.082, p = .125	B = 6.445, p = .106
Site	B = −0.433, p = .518	B = −10.784, p = .192	B = −1.151, p = .833	B = 0.026,p = .827
SES	B = 0.012,p = .571	B = −0.174, p = .519	B = 0.081,p = .921	B = −0.194, p = .958
TCV	B < 0.001, p < 0.001*	B < 0.001, p < 0.001*	B < 0.001, p < 0.001*	B < 0.001, p < 0.001*
Predictor variables	R AAA	R Central	R Medial	R Cortical
Diagnosis	B = −3.434, p < .001*	B = −1.934, p = .072	B = −1.080, p = .177	B = 0.596, p = .300
Age	B = 0.361, p = .008	B = 0.773, p < 0.001*	B = 0.425, p < 0.001*	B = 0.322, p < 0.001*
Sex	B = 1.828, p = .124	B = 3.116, p = .008	B = 2.107, p = .017	B = 1.349, p = .025
Site	B = −0.265. p = .808	B = −0.228, p = .834	B = 0.160,p = 1.140	B = 0.506, p = .362
SES	B = 0.033,p = .356	B = −0.049,p = .171.	B = −0.070,p = .009	B = −0.020,p = .272
TCV	B < 0.001, p < 0.001*	B < 0.001, p < 0.001*	B < 0.001, p < 0.001*	B < 0.001, p < 0.001*
Predictor variables	R CAT	R Paralaminar		
Diagnosis	B = 0.578, p = .830	B = −1.079, p = .093		
Age	B = 2.217, p < 0.001	B = 0.345, p < 0.001*		
Sex	B = 3.456, p = .244	B = 1.258, p = .075		
Site	B = −1.399. p = .609	B = 0.404, p = .535		
SES	B = 0.047,p = .602	B = 0.006, p = .783		
TCV	B < 0.001, p < 0.001*	B < 0.001, p < 0.001*		

ASD: autism spectrum disorder; L: left hemisphere; AAA: anterior amygdaloid area; SES: socioeconomic status; TCV: total cerebral volumes; CAT: cortico-amygdaloid transition area; R: right hemisphere.

* p < 0.002.

### Amygdala subnuclei volumes and anxiety

The total SCARED-P scores were significantly predicted by right basal (B = 0.235, p = 0.002) and right paralaminar (B = −.99, p = 0.009) volumes. Diagnosis (B = 11.95, p < 0.001) and male sex were both significant predictors in the model (B = −5.06, p = 0.006) (Table 4).

**Table 4.** Generalized linear model results for anxiety measures.

Predictor variables	SCARED-P	SDQ-Internalizing	SCARED-SR	CBCL-AD
Diagnosis	B = 11.95, p < 0.001*	B = 4.84, p < 0.001*	B = 8.769, p < 0.001*	B = 9.02, p < 0.001*
Age	B = −0.067, p = 0.774	B = 0.158, p = 0.017	B = −0.512, p = 0.147	B = −0.094,p = 0.587
Sex	B = −5.06, p = 0.006*	B = −0.96, p = 0.041	B = −2.898, p = 0.247	B = −2.19, p = 0.076
Site	B = 3.83, p = 0.015	B = 1.17, p = 0.007*	B = 3.287, p = 0.144	B = 0.735,p = 0.508
SES	B = −0.096,p = 0.079	B = −0.037,p = 0.014	B = −0.119, p = 0.133	B = −0.007, p = 0.862
TCV	B < 0.001, p = 0.038	B < 0.001, p = 0.759	B < −.001, p = 0.234	B < 0.001, p = 0.862
L lateral	B = 0.001, p = 0.658	B = 0.002, p = 0.684	B = 0.014,p = 0.653	B = 0.005, p = 0.751
L basal	B = −0.112, p = 0.177	B = −0.065,p = 0.004*	B = −0.181, p = 0.129	B = −0.066,p = 0.247
L accessory basal	B = 0.171, p = 121	B = 0.072,p = 0.017	B = 0.178, p = 0.233	B = 0.057,p = 0.571
LAAA	B = 0.144, p = 0.415	B = −0.02, p = 0.664	B = 0.183, p = 0.463	B = −0.029,p = 0.819
L Central	B = 0.097,p = 0.493	B = 0.046,p = 0.231	B = 0.099,p = 0.620	B = 0.057,p = 0.571
L Medial	B = −0.209, p = 0.31	B = −0.028,p = 0.628	B = −0.279, p = 0.305	B = −0.018,p = 0.226
L Cortical	B = −0.235, p = 0.501	B = −0.097,p = 0.339	B = −0.102, p = 0.832	B = −0.254, p = 0.332
LCAT	B = −0.501, p = 0.532	B = −0.013,p = 0.574	B = −0.006, p = 0.956	B = −0.018,p = 0.765
L Paralaminar	B = 0.19, p = 0.617	B = 0.272, p = 0.008*	B = 0.57, p = 0.281	B = 0.160, p = 0.549
R lateral	B = −0.004, p = 0.876	B = −0.002, p = 0.788	B = −0.014,p = 0.659	B = −0.006, p = 0.707
R basal	B = 0.235, p = 0.002*	B = 0.061, p = 0.003*	B = 0.148, p = 0.168	B = 0.082, p = 0.134
R accessory basal	B = −0.187, p = 0.069	B = 0.063, p = 0.024	B = −0.243, p = 0.092	B = −0.094, p = 0.188
R AAA	B = −0.299, p = 0.073	B = 0.03, p = 0.502	B = −0.096, p = 0.673	B = −0.008, p = 0.943
R central	B = 0.166, p = 0.30	B =−0.015, p = 0.730	B = 0.193, p = 0.361	B = 0.136, p = 0.217
R medial	B = 0.105, p = 0.583	B = 0.041, p = 0.444	B = −0.001, p = 0.996	B = 0.011, p = 0.935
R cortical	B = −0.026, p = 0.942	B = −0.029, p = 0.766	B = 0.293, p = 0.543	B = −0.029, p = 0.911
R CAT	B = −0.032, p = 0.704	B =−0.029, p = 0.766	B = 0.065, p = 0.559	B = 0.027, p = 0.652
R Paralaminar	B = −0.99, p = 0.009*	B = −0.257, p = 0.013	B = −0.546, p = 0.297	B = −0.272, p = 0.315

Results from generalized linear models investigating the association between Anxiety Scores (SCARED-P, SDQ-Internalizing, SCARED-SR, and CBCL-AD: dependent variables) and diagnosis (independent variable) with age, biological sex, testing site, and SES as covariates. Beta values and p values are shown. The alpha level has been adjusted for multiple comparisons. SCARED-P: Screen for Child Anxiety Related Disorders–Parent Report; SDQ: Strengths and Difficulties Questionnaire Internalizing subscale; SCARED-SR: Screen for Child Anxiety Related Disorders–Self-Report; CBCL-AD: Child Behavior Checklist–Anxious Depressed Subscale; SES: socioeconomic status; TCV: total cerebral volume; L: left hemisphere; AAA: anterior amygdaloid area; CAT: corticoamygdaloid transition area; r: right hemisphere.

* p < 0.0125.

For SDQ-Internalizing scores, significant relationships were found with left (B = −.065, p = 0.004) and right (B = 0.061, p = 0.003) basal volumes, as well as with left paralaminar (B = 0.272, p = 0.008) volumes. Following Bonferroni correction for multiple comparison, right paralaminar (B = −.257, p = 0.013) and left accessory basal (B = 0.072, p = 0.017) volumes were not significant. Diagnosis was significant (B = 4.84, p < 0.001), as was data collection site (B = 1.17, p = 0.007) (Table 4).

Subnuclei volumes were not significantly associated with SCARED-SR scores (all, p > 0.05) nor with CBCL-AD scores (all, p > 0.05).

We conducted a collinearity analysis of the data using linear regression models for the anxiety measures (SCARED-P/SR, SDQ-Int) that were significantly associated with the subnuclei volumes. A third of the nuclei (33%) had variance inflation factors > 10. Mainly these were the nuclei complex that make up the BLA, which is to be expected as they are from a similar nuclei complex. Removing these subnuclei from the analyses produced comparable results.

To determine whether participants with comorbid anxiety diagnoses influenced amygdala subnuclei models, we ran the full model GLMs investigating diagnosis and subnuclei volumes on the four anxiety measures with these 12 participants omitted, and results were maintained. The same amygdala subnuclei were significantly associated with anxiety for the SCARED-P and SDQ-Internalizing measures, and no volumes were associated with anxiety for the CBCL-AD and SCARED-SR measures. No association between whole amygdala volumes and the anxiety measures was evident (SCARED-P: B = 0.001, p = 0.8; SCARED-R: B = −0.001, p = 0.8; CBCL-AD: B = −0.0003, p = 0.89; SDQ-Int: B = −0.00007, p = 0.9).

**Figure 3.**
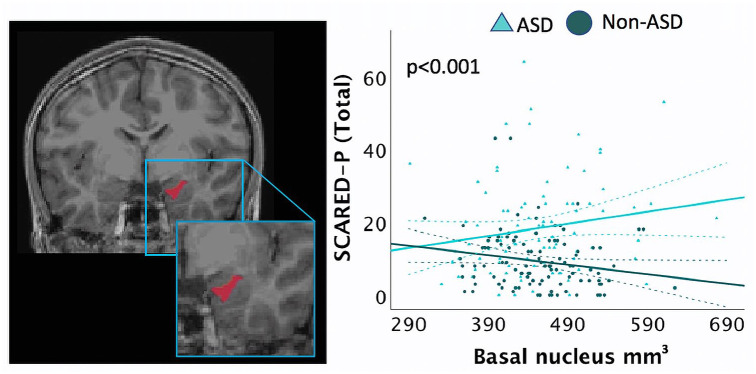
Screen for Child Anxiety Related Disorders (SCARED) scores in an interaction analysis for group (ASD, non-ASD× right basal nuclei volumes, adjusting for age, biological sex, SES, data collection site, and total cerebral volume (TCV). Children with ASD with larger basal nuclei volumes had higher SCARED-P scores. Dashed lines represent 95% confidence intervals.

A GLM was run to investigate the interaction between diagnostic groups (ASD, non-autistic) and right basal and right paralaminar volumes, in predicting SCARED-P scores, with biological sex included as a covariate (Table 5). Results revealed a significant association between ASD group and right basal volumes (B = 0.165, p < 0.001) and between ASD group and right paralaminar volumes (B = −1.301, p = 0.004) for the SCARED-P scores. Within the ASD group, larger right basal volumes (Figure 3) and smaller paralaminar volumes predicted higher SCARED-P scores.

**Table 5.** Results from generalized linear models investigating the interaction effects for amygdala subnuclei volumes in relation to diagnosis for SCARED-P Anxiety Scores (A) and SDQ-Internalizing Anxiety Scores.

Predictor variables	SCARED-P
Sex	B =−4.029, p = 0.017*
R basal × Diagnosis (ASD)	B = 0.165, p < 0.001*
R basal × Diagnosis (non-ASD)	B =−0.004, p = 0.928
R PL × Diagnosis (ASD)	B =−1.301, p = 0.004*
R PL × Diagnosis (non-ASD)	B =−0.002, p = 0.996
Predictor variables	SDQ-Internalizing
Site	B = 0.971, p = 0.028
L basal × Diagnosis (ASD)	B =−0.006, p = 0.746
L basal × Diagnosis (non-ASD)	B =−0.018,p = 0.216
L PL × Diagnosis (ASD)	B = 0.005, p = 0.962
L PL × Diagnosis (non-ASD)	B = 0.056,p = 0.599
R basal × Diagnosis (ASD)	B = 0.009, p = 0.373
R basal × Diagnosis (non-ASD)	B = 0.006, p = 0.549

Results from generalized linear models investigating the interaction effects between SCARED-P Anxiety Scores (A) and SDQ-Internalizing Anxiety Scores (Beta values and p values are shown). The alpha level has been adjusted for multiple comparisons. SCARED-P: Screen for Child Anxiety Related Disorders–Parent Report; SDQ: Strengths and Difficulties Questionnaire Internalizing subscale; R: right hemisphere; ASD: autism spectrum disorder; L: left hemisphere; PL: paralaminar nucleus.

* p < 0.025.

A final GLM was run examining interaction effects between the right and left basal and left paralaminar nuclei volumes and diagnostic groups in predicting SDQ-Internalizing scores, with data collection site included as a covariate (Table 5). No significant interaction effects were found.

## Discussion

Our current study aimed to address two key questions: (1) do children with ASD experience elevated anxiety levels compared with non-autistic children?; (2) is there an association between amygdala subnuclei volumes and anxiety in children with ASD? Using structural MRI and standardized parent and self assessments of anxiety in children and youth, we report that participants with an ASD diagnosis display elevated anxiety on all anxiety measures when compared with non-autistic participants. Participants displayed significant differences in macrostructural association of the amygdala subnuclei between diagnostic groups. Amygdala nuclei volumes were associated with anxiety scores. The right basal nucleus and right paralaminar nucleus were found to be significantly associated with increased anxiety in the ASD group.

### Anxiety in children and youth

Consistent with previous literature, findings from our current study revealed heightened anxiety in the ASD group compared with the non-autistic group of participants, as measured by the parent and self-report SCARED, the CBCL anxiety/depression subscale, and the internalizing scale of the SDQ. These results support our current understanding of anxiety in the ASD population. We analyzed four different anxiety measures: SCARED-P, SCARED-SR, CBCL-AD, and SDQ-Internalizing, and all were sensitive to differences in anxiety exhibited by children with ASD compared with non-autistic children. The SCARED-P and SR has been found to accurately report anxiety in both ASD and non-autistic children (Birmaher et al., 1999; Stern et al., 2014) while other measures may capture different psychometric properties in non-autistic and ASD populations. Many current anxiety instruments are based on age-dependent manifestations of anxiety, which may not reflect anxiety in ASD participants (White et al., 2009). Future studies should investigate the differences that exist between parent and self-report measures of anxiety in subsequent behavioral and neurological comparisons between ASD children and non-autistic children. Distinct anxiety subtypes may also reveal differential amygdala volumetric associations in children. Indeed, increased amygdala volumes were found in children with social anxiety disorders alone, and comorbid generalized anxiety disorder, when compared with non-autistic children (Suor et al., 2020). Children with PTSD have shown maturational variation in the amygdala, with increased volumes in younger individuals, and decreased volumes in older individuals (Weems et al., 2013). Future work should continue to investigate possible amygdala subnuclei associations with specific anxiety disorders in ASD populations.

### Amygdala volumes and anxiety in ASD

Amygdala subnuclei volumes were individually examined with their relationship to participant biological sex, age, diagnosis, and covaried for data collection site, and TCV. Prior to the correction for multiple comparisons, right basal nuclei and AAA volumes were all found to differ between ASD and non-autistic participants, as did left AAA volumes.

Each anxiety measure was independently examined to identify which variables were significantly associated with increased anxiety scores. Right basal and paralaminar volumes were associated with SCARED-P scores. For participants with ASD, a significant diagnosis by right basal volume interaction was found for the SCARED-P, with larger volumes in ASD participants associated with higher anxiety scores, while the same relationship was not seen in the non-autistic group. A similar significant interaction effect was seen with diagnosis and right paralaminar volumes. There was a significant association between smaller paralaminar volumes and SCARED-P anxiety scores in the ASD group, while no significant relationship was seen in the non-autistic group.

Bilateral basal and left paralaminar volumes significantly contributed to SDQ-Internalizing scores. No significant interaction effects between diagnosis and these volumes were found for the SDQ-Internalizing scores. Amygdala nuclei volumes were not significantly associated with CBCL-AD or SCARED-SR scores.

In the current study, basal nuclei volumes were associated with greater SCARED-P and SDQ-Internalizing anxiety scores. The basal nucleus of the amygdala receives sensory information from the lateral nucleus, as well as projections from the OFC and the PFC (Davis & Whalen,2001), suggesting it is heavily involved in emotional regulation (Asami et al., 2018) and is necessary for typical responses to stimuli in learning and memory processing (Baxter & Murray, 2002). Reciprocal connections from the basal nucleus, within the BLA complex, to the PFC and OFC (Felix-Ortiz et al., 2016) indicate that dysfunction to this region, such hyperexcitability, could result in downstream effects to social cognition (Sinha et al., 2015). Larger whole amygdala volumes have been associated with increased anxiety and depression in ASD (Juranek et al., 2006). It has been postulated that chronic stress causes BLA neurons to become hyperexcitable (Sharp, 2017). The larger volumes of basal nuclei seen in the ASD participants may indicate increased excitation of these nuclei (Qin et al., 2014). Chronic stress may be experienced by individuals with ASD in school and social contexts, and as such hyperexcitability of the basal nucleus may be underlying the increased anxiety seen in this population.

Smaller paralaminar nuclei volumes were associated with higher anxiety scores on both the SCARED-P and the SDQ-Internalizing. The paralaminar nucleus is a small and understudied amygdala nucleus in humans. This nucleus contains a high proportion of immature neurons, which are thought to migrate to other nuclei during development and beyond (Sorrells et al., 2019). Adult military veterans suffering from post-traumatic stress disorder have displayed smaller paralaminar volumes, which suggests this particular nucleus may be susceptible to trauma-inflicted damage (Morey et al., 2012). While these previous findings are from an older adult population, our findings are aligned with the results and may indicate this nucleus may be associated with the adverse effects of stress.

### Amygdala volumes and ASD

Bilateral AAA volumes significantly differed between the two participant groups, suggesting these nuclei contribute to ASD symptomatology rather than anxiety. In rodents, the AAA nucleus receives olfactory and other sensory input (Cádiz-Moretti et al., 2017). Cholinergic neurons in the AAA receive direct outputs from the Ce and are believed to be implicated in attention and vigilance (Gastard et al., 2002). While more research is needed into the contributions of the AAA to human behavior, it is possible these nuclei are associated with heightened sensory processing common in individuals with ASD.

ASD in young children has been associated with larger amygdala volumes (Munson et al., 2006; Nordahl et al., 2012). Further investigations have reported an overgrowth of the amygdala in infants and toddlers with ASD (Avino et al., 2018; Li et al., 2019), with adult amygdala sizes reached during childhood, after which amygdala growth plateaus (Schumann et al., 2004). In contrast, non-autistic individuals display amygdala growth throughout infancy and childhood, with growth ceasing during late adolescence (Ostby et al., 2009; Schumann et al., 2004; Wierenga et al., 2020). Some research has reported greater total amygdala volumes in non-autistic adults than ASD adults (Nacewicz et al., 2006) with other studies finding no significant total amygdala volume differences between ASD and non-autistic adults (Xu et al., 2020). Our participants’ ages spanned from childhood to late adolescence, thus future studies are needed to investigate whether the nucleic volume and anxiety relationships we report remain evident in adult populations. Sex differences in amygdala volumes have also been reported in children with ASD (Walsh et al., 2021). In the current work, sex differences were found for the AAA volumes. Our sample did not include a large number of female participants. Future work in larger samples of males and females with ASD to examine sex differences in amygdala subnuclei volumes would be warranted.

### Limitations

Reliable anxiety measures, including the SDQ, SCARED, and CBCL-AD, may be confounded by existing ASD symptomatology (van Steensel et al., 2011). Attempting to distinguish the two may be an especially important consideration for future studies examining neurological correlates of anxiety symptoms in ASD. In addition, individuals with ASD may display atypical manifestations of anxiety which are not captured by current standardized anxiety measures. Children with ASD with higher IQs may also exhibit increased anxiety (Mingins et al., 2021). This may lead to differences in anxiety being reported in ASD participants. Recently, autism-specific anxiety assessments have been developed which are designed to accurately measure anxiety symptoms in individuals with autism by clearly differentiating overlapping symptoms (Kerns et al., 2017a). Examples of such assessments include the Anxiety Disorders Interview Schedule–Autism Addendum (Kerns et al., 2017b) the Parent-Rated Anxiety Scale for Youth with Autism Spectrum Disorder (Scahill et al., 2019), and the Anxiety Scale for Children with Autism Spectrum Disorder (Rodgers et al., 2016). Future studies assessing anxiety in children with ASD should utilize these measures which may better quantify anxiety symptoms in the ASD population.

ASD is a male-biased disorder with a current male to female ratio of 4:1 (Ali et al., 2022; Davidovitch et al., 2013; Hinkka-Yli-Salomäki et al., 2014; Taylor et al., 2013). Our current study reported a significant association between sex and SCARED-P scores, with higher anxiety reported by females. Our study was not poised to further investigate sex differences and the associations between amygdala subnuclei volumes and anxiety in ASD, and we encourage that future research should be focused on this important issue.

Age was included as a covariate in our statistical models and was significantly associated with nuclei volumes, but not anxiety. As a cross-sectional study, the ability to further explore the effects of age on anxiety and amygdala nuclei volume in children with ASD. A previous report indicated that children with higher anxiety had smaller amygdala volumes; however, the association between amygdala volume and anxiety was less prominent at older ages (Warnell et al., 2018). Future longitudinal studies are necessary to determine whether anxiety follows similar age-related trajectories in children and adolescents with ASD as non-autistic youth.

## Conclusion

We report that children with ASD experienced higher anxiety assessed via parent and self-report compared with non-autistic children. Larger basal nuclei volumes were predictive of anxiety in children and adolescents with ASD. Notably, results demonstrate differences between amygdala subnuclei, indicating the clinical significance of evaluating separate contributions of amygdala substructures in anxiety in children with ASD. Future research on a microanatomical level is needed to investigate amygdala subnuclei structure in ASD and the association with mental health outcomes. This research also highlights the need for longitudinal research in this area to examine changes over time in amygdala subnuclei development in relation to long-term assessments of anxiety in children with ASD.
